# Bicycle helmet use and non-use – recently published research

**DOI:** 10.1186/1745-6673-7-9

**Published:** 2012-05-25

**Authors:** Stefanie Uibel, Daniel Müller, Doris Klingelhoefer, David A Groneberg

**Affiliations:** 1 Institute of Occupational, Social and Environmental Medicine, Goethe-University, Theodor-Stern-Kai 7, 60590, Frankfurt am Main, Germany

**Keywords:** Bicycle helmet, Protection, Prevention, Accident, Trauma

## Abstract

Bicycle traumata are very common and especially neurologic complications lead to disability and death in all stages of the life. This review assembles the most recent findings concerning research in the field of bicycle traumata combined with the factor of bicycle helmet use. The area of bicycle trauma research is by nature multidisciplinary and relevant not only for physicians but also for experts with educational, engineering, judicial, rehabilitative or public health functions. Due to this plurality of global publications and special subjects, short time reviews help to detect recent research directions and provide also information from neighbour disciplines for researchers. It can be stated that to date, that although a huge amount of research has been conducted in this area more studies are needed to evaluate and improve special conditions and needs in different regions, ages, nationalities and to create successful prevention programs of severe head and face injuries while cycling.

Focus was explicit the bicycle helmet use, wherefore sledding, ski and snowboard studies were excluded and only one study concerning electric bicycles remained due to similar motion structures within this review. The considered studies were all published between January 2010 and August 2011 and were identified via the online databases Medline PubMed and ISI Web of Science.

## Objective

Bicycle traumata are common causes of death and disability in all ages with a gravity of severe neurologic injuries in children and adolescents. Correctly worn bicycle helmets can reduce fatal and non-fatal head and brain injuries – these facts seem to be evidently and confirmed by a multitude of prior and recent studies. Unclear remains the range of benefits in protection of other injuries due to traumatic bicycle accidents as well as sufficient strategies of implementing the gained scientific knowledge into daily habit routines. Diverse studies measured also differences of attitude and behaviour in different person groups as well as in regions with or without legal regulations concerning helmet use. Due to the interdisciplinary field between prevention, acute medicine, epidemiology and legislation, scientists are barely able to monitor all disciplines. Therefore this article should provide insights into the ongoing research in the plurality of global publications and research projects concerning bicycle helmet use.

## Design

In order to analyse the most recent findings concerning research in the field of bicycle traumata and the cofactor bicycle helmet use, search termini were selected and a search within the online databases Medline PubMed including the use of the MeSh-database as well as searching through ISI Web of Science was conducted. The screened studies cover the time span between January 2010 to August 2011, reviews, letters or case reports were excluded.

## Results

Due to the multidisciplinary field of research, the studies are sorted under the following categories (see Table 1): medical findings, passive transportation on bicycles, co-factors, educational efforts and prevention, special terrain, region specific analyses, meta-analysis concerning helmet efficacy. No studies concerning technical or ergonomical helmet improvements were identified in the here considered time period.

**Table 1:** Overview -- Bicycle helmet use and non-use

**Category**	**Study focus [citation]**
1. medical findings	mandibular condylar fractures [1]
	dentoalveolar traumata [2]
	also see region specific analyses [3]: characteristics and outcome of bicycle injuries in paediatric patients
2. passive transportation on bicycles	dummy study on infants in bicycle-mounted child seats [4]
3. co-factors	model analyses of crashes at intersection and non-intersection locations [5]
	depressional symptoms and health-related risk-taking behaviours during adolescence [6]
	alcohol use in correlation with head injury and helmet use [7]
4. educational efforts and prevention	depiction of injury-prevention practices in children’s movies [8]
	effectiveness of a bicycle software program [9]
	attitude of neurosurgeons concerning helmet use [10]
	prediction of helmet use among undergraduates by helmet attitudes scale and health belief model [11]
5. special terrain	mountain bike terrain park injuries [12]
6. region specific analyses	Germany [13]
	Canada [14-17]
	Spain [18]
	USA [19,20]
	Hungary [3]
	China [21]
7. meta-analyses	re-analyzed meta-analysis data from 2001 of Attewell, Glase and McFadden [22]

### Medical findings

Two retrospective studies analyzed injury characteristics depending on helmet usage or non usage.

Sawazaki et. al. focussed retrospectively on epidemiologic characteristics of prevalence, incidence and treatments of mandibular condylar fractures in their Brazilian study [1]. The data collection covered the years 1999 – 2007 and included facts about demographic data, aetiology, diagnosis, type, dislocation, use of protective devices, state of the dentition, associated facial and general trauma, soft tissue lesions, treatment methods as well as the interval between trauma and treatment. Within the total of 317 fractures (209 unilateral and 54 bilateral fractures, male/female ratio of 3.05:1, mean age of 28.4 years), the most common causes were road traffic accidents (57.8%), in which young adults were involved [1]. An important finding was the significantly decreased number of bilateral condylar fractures occurring from road traffic accidents (P < .05) due to protective devices as seatbelts and helmets [1]. Beside other results they conclude clearly that mandatory use of safety helmets and seatbelts are together with intensified educational efforts essential to decrease the number of facial fractures.[1]

Also in a 9-year retrospective study Santos et. al. analyzed the occurrence of sustained oral and maxillofacial traumatic injuries associated with dentoalveolar trauma in the Oral and Maxillofacial Surgery Division at Piracicaba Dental School, State University of Campinas, Sao Paulo, Brazil [2]. The data set included information about age, gender, aetiology, use of protective devices such seatbelts or crash helmets, and presence of facial fractures and general trauma, oral condition, stage of dentition, date of trauma, drug abuse, type, teeth affection and classification of the trauma [2]. A total of 2,785 patients were admitted with facial traumata and 542 (19.46%) showed dental and dentoalveolar traumata. Out of these 542 analyzed patients (male/female ratio 2.81:1, 79.34% in the first three decades) smoking was identified as the most common harmful habit (16.05%) followed by alcohol use (15.87%) [2]. Furthermore were bicycle accidents (26.94%) the most common cause, followed by falls (22.69%), wherein 31.51% of drivers were wearing seatbelts and 84.38% of motorcycle drivers were wearing helmets at the moment of injury [2]. Santos et.al. also found, that the weekends were the periods with the major incidence of dentoalveolar trauma as well as alcohol consumption was linked with this type of trauma [2]. The authors indicated that their study could be biased, because most dentoalveolar injuries were more frequent be managed in general dental, pediatric dental or oral surgical offices and were not carried into an emergency department unless the injuries were classified as more severe or unusual.[2]

### Passive transportation on bicycles

In a setting of biometrical dummy tests Miyamoto et.al. aimed to confirm in their study the risk of bicycle-mounted child seats and to evaluate the efficacy of helmets, seat belts and back seat height in their study in terms of preventing contact-type head impacts that occur in falls from bicycle-mounted child seats. [4] Methodically anthropometric test dummies were placed in stationary bicycles within a bicycle-mounted child seat and were tipped over in repetition. Head Injury Criteria were calculated, where the main finding was, that only helmets lowered explicit the maximal acceleration and the head injury severity with statistical significance.[4] The lowest injury scores were measured when the dummy wore both a helmet and a seat belt sitting in a high-back seat. Miyamoto et.al. summed up and highly recommended, that only the combination of bicycle helmet, seat belt and especially a high enough seat back could protect a children’s head from a contact-type injury.[4]

### Co-factors

Moore et. al. published recently a retrospective study concerning model analyses of bicyclist injury severity resulting from motor vehicle crashes at intersection and non-intersection locations.[5] They developed standard multinomial logit and mixed logit models to estimate the degree of influence that bicyclist, driver, motor vehicle, geometric, environmental and crash type characteristics have on bicyclist injury severity. [5] Classifications for the severity were used as property damage only, non incapacitating or incapacitating. Their study based on 10,029 bicycle involved crashes that occurred in the State of Ohio, USA, from 2002 to 2008 and presented via analyses of likelihood ratio tests that some of the factors affecting bicyclist injury severity at intersection and non-intersection locations were substantively different. [5] This implicated their development of separate models to independently assess the impacts of various factors on the degree of bicyclist injury severity resulting from crashes at intersection and non-intersection locations.[5] Moore et. al. found in their work, that several covariates had similar impacts on injury severity at both intersection and non-intersection locations, but contrariwise, six variables were found to significantly influence injury severity at intersection locations but not non-intersection locations while four variables influenced bicyclist injury severity only at non-intersection locations.[5] Concerning accidents at intersection locations the probability of an incapacitating, severe bicyclist injury found to be increased by 14.8% if the bicyclist was not wearing a helmet, 82.2% if the motorist was under the influence of alcohol, 141.3% if the crash-involved motor vehicle was a van, 40.6% if the motor vehicle striked the side of the bicycle, and 182.6 percent if the crash occurred on a horizontal curve with a grade.[5] Moreover, the study showed, that the likelihood of severe injuries in non-intersection areas increased by 374.5% if the bicyclist was under the influence of drugs, 150.1% if the motorist was under the influence of alcohol, 53.5% if the motor vehicle striked the side of the bicycle and 99.9% if the crash-involved motor vehicle was a heavy-duty truck.[5]

Another group, Testa et.al., investigated via a survey of 20,745 adolescents (data from the National Longitudinal Study of Adolescent Health provided) the relation between depressive symptoms and a variety of health-related risk-taking behaviours during adolescence.[6] They could show, that people who reported more depressive symptoms as well as levels of hopelessness were found to wear seatbelts less often, wear bike-helmets less often, and drive while drunk more frequently.[6] No correlation could be shown with the reported use of condoms. To conclude, Testa et. al. found in adolescents suffering from depressive symptoms and here especially those reporting hopelessness, a high risk potential for a multitude of health-related risk-taking behaviours like the here focussed non-use of helmets.

Crocker and colleagues from Austin, Texas, USA, aimed to examine the interactions between alcohol, bicycle helmet use, experience level, riding environment, head and brain injury, insurance status, and hospital charges in a medium-sized city without an adult helmet law.[7] Methodically they collected data from 200 adult bicycle accident victims presenting to a regional trauma center over a 1-year period (2006–2007) at the bedside in addition to available information concerning prevailing vehicle speed (for road accidents), and presence and degree of head or brain injury.[7] The results showed, that alcohol use was in a strong correlation with head injury (odds ratio, 3.23; 95% confidence interval, 1.57–6.63; *P* = .001). More statistically significant findings (all P values < .05) were, that impaired riders were less experienced, less likely to have medical insurance, rarely wore helmets, were more likely to ride at night and in slower speed zones such as city streets, and their hospital charges were double.[7] The research of Crocker et. al. drawed the conclusions, that alcohol use causes accidents, increasing amounts of head and brain injuries via unsafe bicycling practices and is expensive for the cyclist and community.[7]

### Educational efforts and prevention

The study goal of Tongren et.al. was to determine if the depiction of injury-prevention practices in children’s movies was different from what was reported from 2 earlier studies, which showed infrequent depiction of characters practicing recommended safety behaviours. [8] They examined the top-grossing 25 domestic G-rated (general audience) and PG-rated (parental guidance suggested) movies per year for 2003–2007 under exclusion of movies or scenes that were animated, not set in the present day, fantasy, documentary, or not in English.[8] Injury-prevention practices involving motor vehicles, pedestrians, boaters, and bicyclists were recorded for characters with speaking roles in 76 movies and a total of 958 examined scenes, of which 524 (55%) showed children and 434 (45%) adults. [8] As results, 22 scenes involved crashes or falls, what resulted in 3 injuries and no deaths. The analyzes showed that 311 (56%) of 555 motor-vehicle passengers were belted; 73 (35%) of 211 pedestrians used crosswalks; 60 (75%) of 80 boaters wore personal flotation devices; and 8 (25%) of 32 bicyclists wore helmets.[8] Tongren et. al. stated, that in comparison with previous studies, the usage of safety belts, crosswalks, personal flotation devices, and bicycle helmets increased significantly, which is summed up as the right direction, although approximately fifty percent of the relevant scenes still show unsafe practices and the consequences of these behaviours were rarely shown.[8]

McLaughlin and Glang evaluated in their recent study the effectiveness of the bicycle eHealth software program “Bike Smart” for improving safety-related knowledge and behaviour in 206 elementary students in grades kindergarten under a random control design with students assigned to either the treatment condition (Bike Smart) or the control condition (a video on childhood safety).[9] Their outcome measures included computer-based knowledge items (safety rules, helmet placement, hazard discrimination) and a behavioural measure of helmet placement and the results showed that regardless of gender, cohort, and grade the participants in the treatment group showed greater gains than control participants in both the computer-presented knowledge items (*p* > .01) and the observational helmet measure (*p* > .05). [9] In conclusion, the authors suggest, that the evaluated Bike Smart program could be a low cost, effective component of safety training packages that include both skills-based and experiential training.

The study of Jung et.al. concentrated on the attitude and opinion of neurosurgeons (NS) concerning protective bicycle-helmet use.[10] They pointed out, that although the German Neurosurgical Society advocated compulsory use of bicycle helmets in 2007, the attitude of wearing bicycle helmets remained unclear in the group of neurosurgeons, who are the primarily treating specialists of patients with traumatic brain injuries in Europe.[10] The data collection of Jung et.al. was done via anonymous questionnaires of 55 neurosurgical departments in Germany, Austria, and Switzerland (returned questionnaires n = 465), as control group people of the general public (PUB) were interviewed (returned questionnaires n = 546). As results, 49.7% of the NS and 44.5% of PUB indicated that they wear helmets while bicycling, while trauma experience did effect the personal decision of whether to wear bicycle helmets as well as the support of compulsory use.[10] Furthermore, Jung et.al. found, that NS and PUB behaved in similar ways, because only one half wear protective helmets, while the others show cognitive dissonant behaviour, which showed, that education as well as real life contact did not suffice in promoting the use of bicycle helmets.[10]

A survey study by Ross et. al. developed a bicycle helmet attitudes scale and used the health belief model to predict helmet use among undergraduates.[11] 337 students completed a comprehensive survey on attitudes and behaviours relevant to bicycle helmet use between November 2006 and November 2007. The study results showed that only 12% of the students were self-reported helmet users. The Bicycle Helmet Attitudes Scale scores captured 52% of the variance associated with helmet use; each subscale differentiated wearers from nonwearers, in which for example men reported more media influences than women.[11] The Bicycle Helmet Attitudes Scale contains 57 items and represents 10 reliable subscales and will provide further attempts for promoting cycling safety.[11]

### Special terrains

As an example of bicycle helmet use in special terrains, Ruest et.al. focussed in their prospective case-controlled study on Mountain bike (MB) terrain park injuries as emerging causes of morbidity and determined the injury profile and risk factors for severe injuries among cyclists in MB parks. [12] Definition of cases were hospitalised recreational cyclists injured in MB parks, whereas controls were cyclists injured in MB parks seen and discharged from the four participating, Canadian emergency departments in Calgary (ED).[12] While study time (May 2008 to August 2010) 351 patients were interviewed about risk factors and crash circumstances, injury data was retrieved via medical chart review. The analysis showed, that 23 participants were hospitalised (cases), of which 21% were female (9% of controls). The most common body region injured was the head/neck/face among cases, and the upper extremities among controls. [12] Ruest et.al. also found, that a greater proportion of cases than controls were older than 25 years (22% vs 15%, respectively), that full-face helmets were used less among cases than controls (21% vs 41%, respectively) and that arm and elbow protection was used more among cases than controls (arm: 13% vs 2%; elbow: 22% vs 8%).[12] On univariate analyses, they measured an indication of increased odds of severe injury among females (OR = 2.8; 95% CI 0.96 to 8.06). Furthermore, riding a new bicycle (OR = 2.74; 95% CI 1.16 to 6.45) and cycling on grass compared with dirt (OR = 7.06; 95% CI 1.21 to 41.33) increased the odds of severe injury.[12] The study of Ruest et.al. provided in conclusion data, that suggested surface and experience-related characteristics as increasing risk factors of severe injury as well as revealed differences in protective equipment use in the case–control cohort.[12]

### Region specific analyses

#### Germany

Ritter and Vance research concentrated via a nationwide household survey in Germany (2008) on the investigation of the determinants of voluntary helmet use. [13] Used were a combination of descriptive analyses and econometric methods, especially variants of the probit- and heteroskedastic probit model. [13] Their findings approved prior studies concerning significant correlates of helmet use with household demographics, residential location and riding patterns. Interestingly and contrastingly to other studies, they found that adult women were significantly less likely to use a helmet than men. [13]

#### Canada

Three observational Canadian studies coming from different Institutes of the University of Alberta were recently published:

The field observation study of Hagel and colleagues [15] worked on factors which are associated with incorrect bicycle helmet use. Via two discreet observational surveys conducted in Alberta in 2000 and 2006 information on cyclists including correct helmet use was gathered. [15] In addition, the prevalence of correct helmet use was compared across multiple factors like age, gender, riding companionship, and environmental factors such as riding location, neighbourhood median family income and region. [15] Poisson regression analysis was used to relate predictor variables to the prevalence of incorrect helmet use, adjusting for clustering by site of observation.[15] Observations were made on 9734 cyclists; of these, 5842 (60%) were wearing a bicycle helmet, 20 (0.2%) were wearing another type of helmet and 3872 (40%) were not wearing a helmet.[15] The results showed that 15.3% of the 5862 helmeted cyclists were wearing their helmet incorrectly or were using a non-bicycle helmet. As a prominent finding children (53%) as well as adults (51%) tended to wear their helmet too far back, while adolescents tended to not have their straps fastened (48%).[15] In 76% of the adult cyclists incorrect helmet use was detected over the study period, whereas in children and adolescents approximately 50% showed incorrect helmet use. Hagel et. al. also found, that children were 1.8 times more likely to use their helmets incorrectly in 2000 compared with adults, but this effect increased to 3.9 in 2006.[15] Furthermore Hagel et. al. presented data, that children and adolescents cycling alone were associated with incorrect helmet use, what identified them as high risk groups, who should receive targeted interventions to increase correct helmet use. [15]

Karkhaneh and colleagues recently published two observational studies concerning bicycle helmet use in Canada, focussing once on children and adolescents less than 18 years old [17], and the other time on cyclists of all ages [16]. The first study now investigated bicycle helmet use four years after the introduction of helmet legislation in Alberta, Canada, which was another study comparable to two studies conducted two years before and four years after the introduction of helmet legislation in Alberta in 2002. [17] Bicyclists were observed in randomly selected sites (June to October 2006) and helmet wearing as well as rider characteristics were recorded by trained observers.[17] Poisson regression adjusting for clustering by site was used to obtain helmet prevalence (HP) and prevalence ratio (PR) (2006 vs. 2000) estimates. [17]

Totals of 4002 bicyclists were observed in 2000 and 5365 in 2006 and as results HP increased in all age proportions: from 75% to 92% among children, from 30% to 63% among adolescents and 52% to 55% among adults. Furthermore, Karkhaneh et. al. found in controls for city, location, companionship, neighbourhood age proportion <18, socioeconomic status, and weather conditions, that the helmet use increased 29% among children (PR = 1.29; 95% CI: 1.20–1.39), over 2-fold among adolescents (PR 2.12; 95% CI: 1.75–2.56), and 14% among adults: (PR = 1.14; CI: 1.02–1.27).[17] Overall, the Canadian bicycle helmet legislation was clearly associated with a greater increase in helmet use among the target age group (<18).[17]

In the second published study, Karkhaneh et. al. again observed bicyclists in the above manner from June to September of 2006 in St. Albert, a community subject to both provincial (< 18 years old) and municipal (all ages) helmet legislation in comparison with observational results from 2000 when no legislation existed.[16] Here again HP increased from 45% to 92% (PR = 2.03; 95% CI: 1.72–2.39) post-legislation, while children were 53% (PR = 1.53; 95% CI: 1.34–1.74) and adolescents greater than 6 times (PR =6.57; 95% CI: 1.39–31.0) more likely to wear helmets; but adults (PR = 1.26; 95% CI: 0.96–1.66) did not show a statistically significant change post-legislation.[16]

Also concerning Canadians helmet use and bicycle ridership Dennis et. al. conducted a study focussing on effects of provincial bicycle helmet legislation and on the association between the comprehensiveness of helmet legislation and both helmet use and bicycle ridership.[14] Data of helmet usage was retrieved from the 2005 Canadian Community Health Survey (CCHS), data of bicycle usage was based on data from the 2000–01, 2003, 2005, and 2007 cycles of the CCHS. [14] Whilst bicycle helmet legislation has been variably implemented in six of 10 Canadian provinces, according to the reports helmets were worn by 73.2% (95% CI 69.3% to 77.0%) of respondents in Nova Scotia, where legislation applies to all ages, by 40.6% (95% CI 39.2% to 42.0%) of respondents in Ontario, where legislation applies to those less than 18 years of age, and by 26.9% (95% CI 23.9% to 29.9%) of respondents in Saskatchewan, where no legislation exists.[14] Dennis et. al. found neither changes in recreational and commuting bicycle use nor changes in ridership among youth and adults following to the implementation of legislation in PEI and Alberta, but a significantly increased likeliness to wear helmets as the comprehensiveness of helmet legislation increased.

#### Spain

Research about risk behaviour relationships with road safety in adolescents in Madrid and Andalusia Regions via samples of a cross-sectional descriptive study was provided by Meneses Falcon et.al. [18] In May and June 2007 samples of 3,612 secondary school pupils from Madrid (n = 1708) and Andalusia (n = 1904) were drawn and the data collected included sociodemographic areas (age, sex, grade, father’s profession, birth place) as well as information about the risk situation and behaviour (risk behaviour as driver or passenger).[18] The analyzed findings were inter alia, that 16.2% of the adolescents had been involved in a dangerous situation with motorcycles during the anterior year, whilst 16.7% never used a helmet when riding a motorcycle and 62% did not wear one when riding a bicycle on the road.[18] To continue with the results 17.4% frequently drove a motorcycle over the speed limit and 24.5% a car.[18] Significant differences were found regarding sex, grade and region (Madrid or Andalusia) and also their defined cofactors drugs, speed, security and passenger could be revealed on account of 62% of the erratic behaviours, whereas especially the drug factor (OR = 1.96; 95% CI, 1.77–2.18) and the speed factor ((OR = 2.13; 95% CI, 1.92–2.36) increased the risk of getting into a dangerous situation doubly.[18] Summing up, adolescents in higher grades and living in Andalusia were identified as less road safety conscious, what should be taken into account when designing preventive actions in Road Safety Education. [18]

#### USA

Dellinger and Kresnow compared children’s bicycle helmet use in the USA to that estimated from an earlier study, and explored regional differences in helmet use by existing helmet legislation.[19] Via a cross-sectional, list-assisted random-digit-dial telephone survey, they interviewed 9,684 respondents during 2001–2003, while a subset with at least one child in the household age 5–14 years (2,409 respondents) answered questions about bicycle helmet use for a randomly selected child in their household. [19] Their findings showed that 48% of the children always, 23% sometimes and 29% never wore a bicycle helmet, whereby there existed significant associations with race, ethnicity and child age but was not with the sex of the child.[19] As other significant predictors of use functioned household income, household education, census region and bicycle helmet law status, the latter were in a statewide manner more effective than laws covering smaller areas.[19] In comparison to older data the proportion of children who always wore a helmet increased from 25% in 1994 to 48% in 2001–2002, whilst significant increases in helmet use (20% to 26%) were seen among both sexes, younger (5–9 years) and older (10–14 years) children.[19] The authors could present a substantial progress in the number of children who always wear their helmets, but stated, that nevertheless more than half did not, what should lead to campaigns promoting consistent helmet use.[19]

The next study has to be interpreted according to the authors with caution: As beneficial effects of bicycle helmet use had been reported mostly based on medical or survey data collected from hospitals, the study of Kweon and Lee examined the validity of the United States General Estimates System (GES) database familiar to many transportation professionals.[20] They aimed to prove a potential beneficial effect of helmet use in reducing the severity of injury to bicyclists and to find potential risk of erroneous conclusions that could be drawn by a narrowly focused study when the GES database was used.[20] Using a partial proportional odds model reflecting intrinsic ordering of injury severity about 16,000 bicycle-involved traffic crash records (between 2003 and 2008) in the United States were extracted and analyzed from the GES database.[20] The results showed a beneficial effect of helmet use in 2007, but opposed effects in 2004 and –in contrast to medical or hospital survey data- no effects in 2003, 2005, 2006, and 2008. The variety of results lead the authors to speculate that there might be a possible lack of representation of the GES data for bicycle-involved traffic crashes, which may be supported by the findings, such as the average helmet use rates at the time of the crashes varying from 12% in 2004 to 38% in 2008.[20] Furthermore this suggests that the GES data might not be a reliable source for studying narrowly focused issues such as the effect of helmet use, because of a possible lacks of representation of the GES data.

#### Hungary

Kiss et.al. investigated in their study the characteristics and the outcome of bicycle injuries in paediatric patients according to the living environment and its demographic density and to create guidelines for injury prevention by analyzed hospital acquired data of 1803 in- and out-patient children treated at the Paediatric Surgical Department of Pecs/Hungary (2000–2006), and at the Department of Paediatric Surgery at the Heim Pal Hospital Budapest (2004–2006). [3] Also followed up information were received via mailed questionnaires to the patients’ families. They analyzed three groups according to demographic density (village, midsize town and large town) and found, that poor road quality played an important role as a contributing factor of injuries in villages.[3] Spoke injuries were in the highest amount measured in villages (13%) followed by large towns (9.9%) and midsize towns (4.6%). According to the low use of helmets in villages (5%; about 9% in midsize and large towns) head injuries were more common in villages, whilst in midsize towns and large towns arm injuries proved to be predominant.[3] Kiss et. al. concluded that the identification of health risky behaviour especially in villages showed a need for special attention regarding this higher risk population. [3]

#### China

Feng et.al. published in 2010 their analysis data concerning electric-bicycle-related injuries as a rising traffic injury burden in China based on the Hangzhou Police Bureau’s data on injuries and deaths covering the years 2004–2008.[21] They found that there was a significant average annual increase in electric-bicycle-related casualty rates of 2.7 per 100,000 population (95% CI 1.5 to 3.9, p = 0.005), while at the same time the overall road traffic and manual-bicycle-related deaths and injuries decreased.[21] Since there exist no safety regulations concerning the use of electric bicycles in China, the authors recommended reinforcements of laws and rules and a mandatory helmet use. [21]

### Meta-analyses concerning helmet efficacy

As a very interesting investigation Rune Elvik [22] re-analyzed the meta-analysis data concerning bicycle helmet efficacy from 2001 of Attewell, Glase and McFadden [23], and discovered a publication as well as a time-trend bias. Elvik found, that former suggested positive effects of bicycle helmet use were therefore exaggerated. His re-analysis included four steps: firstly he detected and adjusted for publication bias by means of the trim-and-fill method; secondly Elvik ensured the inclusion of all published studies by means of continuity corrections of estimates of effect rely on zero counts; thirdly he detected and tried to account for a time-trend bias in estimates of the effects of bicycle helmets and fourthly he updated the study by including recently published studies evaluating the effects of bicycle helmets.[22] Summing up the findings Elviks re-analysis showed smaller safety benefits associated with the use of bicycle helmets than the original study.

## Conclusion

**Figure 1:**
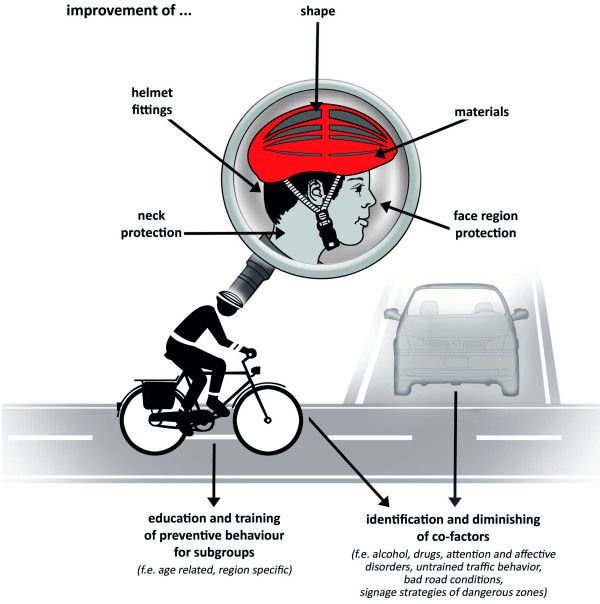
Areas of improvements.

The recently published studies presented a broad variety of approaches dealing with advantages, disadvantages or practices of bicycle helmet use. Due to different legal situations concerning law regularities variant stadiums can interestingly be monitored around the globe. Similarities can be found towards not expedient programs concerning volunteering helmet use and its preventive benefit, although a relative protection of head and brain is stated as secured. The recent studies concerning mandibular condylar fractures and maxillofacial traumatic injuries indicated also a benefit in using helmets [1,2], but studies in greater settings need to be performed to reach distinct and bias-less statistic significance, because helmet benefits for face and neck injuries are up to now only of rarely evidence as Elvik showed [22]. Also especially, but not surprisingly the co-factor alcohol impairs on the one hand the attendance of using protective devices and promotes risky behaviour, on the other hand it worsens the severity and outcome of the listed injuries. New inventions like different airbag protections will surely be important and necessary reopenings especially for people, who object wearing helmets for appearance or stylistic reasons, but their functioning and surety must be tested carefully and for a broader populace they will be at least not affordable. Therefore improvements of the existing devices respectively of the awareness and attitude towards the usage will remain the central fields of research (see Figure 1 Areas of improvements). Since there is a large amount of research data on bicycle helmet use available, future studies applying modern scientometric tools [24-27] should also be performed in this area to provide better insights for physicians and other scientists into this multidisciplinary area of research.

## Competing interests

The authors declare that they have no competing interests.

## Authors’ contributions

SU, DM, DK, DAG have made substantial contributions to the conception and design of the article, acquisition of the data and have been involved in drafting and revising the manuscript. All authors have read and approved the final manuscript.
